# Examining Mental and Physical Health Status Among Sandwich Generation Caregivers

**DOI:** 10.1093/geroni/igab046.1740

**Published:** 2021-12-17

**Authors:** Hansol Kim, Richard Schulz, Scott Beach, Heidi Donovan

**Affiliations:** 1 University of Pittsburgh, Pittsburgh, Pennsylvania, United States; 2 University of Pittsburgh, University of Pittsburgh, Pennsylvania, United States

## Abstract

With a sample of 54,076 caregivers, the Behavioral Risk Factor Surveillance System (BRFSS, 2015-2017) provides an opportunity to assess the impact of caregiving on U.S. adults varying in age, gender, and race. We focused on sandwich generation (SG) caregivers, aged 30-59, with childcare and eldercare responsibilities (n=8,805). In multivariate regression models of mental and physical health, we evaluated the association of age, gender, race and caregiving factors controlling for marital status, education, housing status, income, employment status, and self-rated health status. SG caregivers were predominantly female (65.6%), white (69.6%), black (13%) and were caring for a parent/parent-in-law (50%) or another relative (26.4%). Age, gender, and race were associated with mental health outcomes such that SG caregivers who were younger (aged 30-39), female, and white reported poorer mental health during the past month (p<.05). Older (aged 50-59), female, and white SG caregivers reported poorer physical health during the past month (p<.05). Caregiving factors were not associated with mental health, but SG caregivers who had been providing care for a longer period of time were more likely to report poor physical health in the past month. Finally, education, income, employment, housing status, and self-rated health status were statistically significant covariates in both models (p<.05). These findings demonstrate that young caregivers are at risk for poor mental health; older caregivers for poor physical health. Being female, white, with lower socioeconomic status is associated with poor mental and physical health. Future research should address the unique needs of SG caregivers with dual caregiving responsibilities.

